# Improving Self-Help E-Therapy for Depression and Anxiety Among Sexual Minorities: An Analysis of Focus Groups With Lesbians and Gay Men

**DOI:** 10.2196/jmir.4013

**Published:** 2015-03-11

**Authors:** Tomas Rozbroj, Anthony Lyons, Marian Pitts, Anne Mitchell, Helen Christensen

**Affiliations:** ^1^Australian Research Centre in Sex, Health and SocietyLa Trobe UniversityMelbourneAustralia; ^2^Black Dog InstituteSydneyAustralia

**Keywords:** Internet therapy, e-therapy, cCBT, mental health, gay men, lesbian, minority stress, depression, anxiety, focus groups

## Abstract

**Background:**

E-therapies for depression and anxiety rarely account for lesbian and gay users. This is despite lesbians and gay men being at heightened risk of mood disorders and likely to benefit from having access to tailored self-help resources.

**Objective:**

We sought to determine how e-therapies for depression and anxiety could be improved to address the therapeutic needs of lesbians and gay men.

**Methods:**

We conducted eight focus groups with lesbians and gay men aged 18 years and older. Focus groups were presented with key modules from the popular e-therapy “MoodGYM”. They were asked to evaluate the inclusiveness and relevance of these modules for lesbians and gay men and to think about ways that e-therapies in general could be modified. The focus groups were analyzed qualitatively using a thematic analysis approach to identify major themes.

**Results:**

The focus groups indicated that some but not all aspects of MoodGYM were suitable, and suggested ways of improving e-therapies for lesbian and gay users. Suggestions included avoiding language or examples that assumed or implied users were heterosexual, improving inclusiveness by representing non-heterosexual relationships, providing referrals to specialized support services and addressing stigma-related stress, such as “coming out” and experiences of discrimination and harassment. Focus group participants suggested that dedicated e-therapies for lesbians and gay men should be developed or general e-therapies be made more inclusive by using adaptive logic to deliver content appropriate for a user’s sexual identity.

**Conclusions:**

Findings from this study offer in-depth guidance for developing e-therapies that more effectively address mental health problems among lesbians and gay men.

## Introduction

E-therapies are programs that utilize the Internet or mobile phones to deliver interactive interventions for preventing and treating depression, anxiety, and other mental health problems. E-therapies most commonly utilize cognitive behavioral therapy (CBT) [1-3], are typically undertaken over several weeks or months, and involve users completing modules or exercises while receiving feedback on their progress. E-therapies show considerable clinical benefits, especially in the treatment of depression and anxiety [2,4,5], and have become an increasingly important part of strategies to address mood disorders in a number of countries, such as Australia [1,6], United Kingdom [7], and New Zealand [8].

An important advantage of e-therapies is their accessibility, especially for marginalized populations [9,10], such as lesbians and gay men [11,12]. Same-sex attracted people often report stigma and other negative experiences as barriers to accessing traditional health care [13-15]. When they do access treatment, they are also less likely than the general population to report feeling satisfied [13,16]. E-therapies have potential to resolve these issues, as they are accessible from anywhere, anonymous, and typically available for free or at low-cost, thus making them an attractive alternative to face-to-face therapy. Furthermore, e-therapies have potential for delivering tailored content to address specific issues faced by lesbians and gay men, such as “coming out”, or disclosing their sexual orientation to family, friends, and co-workers, as well as managing experiences of discrimination and the challenges of engaging in stigmatized same-sex relationships.

Improving access to mental health care is further warranted given that same-sex attracted populations have disproportionately high rates of depression and anxiety [13,17-20], which are largely the result of chronic stress that arises from ongoing experiences of stigma, discrimination, and marginalization [21,22]. In Australia, the 2007 National Survey of Mental Health and Well-Being found that same-sex attracted people were two times more likely to experience anxiety and three times more likely to experience depression [23]. In the United Kingdom, the 2012 Stonewall Report found 22% of gay and bisexual men experienced moderate to severe depression, compared to 9% of the population in general [14,18]. In the United States, there is considerable variance between states, but a 2014 American Psychological Association (APA) report concluded that lesbian, gay, and bisexual people are twice as likely on average to report a mental health disorder [17]. A range of studies from a host of countries also shows that lesbians and gay men are at increased risk of substance abuse problems [13,24] and suicide [18,25].

Despite potential for addressing mental health issues among lesbians and gay men, e-therapies currently fail to cater to these populations. Organizations such as the National Health Service (United Kingdom) [15], Beyondblue (Australia) [26], and the World Health Organization [27,28] are advocating for mental health responses that specifically address the needs of non-heterosexual people. However, a recent review of 24 Web- and mobile phone-based e-therapies found only a few e-therapies included any content to address the specific experiences of being same-sex attracted, such as coming out, same-sex relationships, or stigma-related challenges [29]. Less than a fifth (17%) had gay or lesbian-specific mental health referrals such as helplines, and more than half (58%) used language and examples that assumed or suggested users were heterosexual. Thus, currently available e-therapies may not be as effective as they could be for lesbians and gay men, who may not relate to or may even feel alienated by content that appears designed for heterosexual audiences [29].

To inform the development of tailored e-therapies, we explored possible ways in which e-therapies could be tailored to better cater to the needs of lesbians and gay men. We conducted eight focus groups involving lesbians and gay men with the aim of identifying key areas in which e-therapies could be improved to make them more inclusive, relevant, and appealing to lesbians and gay men. It is important to note that many of the findings reported in this article may also apply to other non-heterosexual populations, such as bisexual men and women. However, these and other sexual identity groups face challenges that are often different from those of lesbians and gay men [30,31], and therefore merit separate studies. It was unfortunately beyond the scope of this study to include all sexual identity groups, so we have focused on lesbians and gay men as a starting point.  

## Methods

### Sample

The sample comprised 32 participants, consisting of 14 women and 18 men, and an average of four participants per focus group. All participants indicated that they were lesbian or gay and were aged 18 years or older. A total of 15 participants were aged 35 years or older and 17 were aged between 18 and 34 years. The mean participant age was 34 years (SD 15). The sample was highly educated on average, with 20 reporting having a university degree. At the start of each focus group, participants were asked to indicate their level of knowledge of e-therapies on a five-point scale (1: I have no knowledge at all, 2: I have very little knowledge, 3: I have moderate knowledge, 4: I have good knowledge, 5: I have excellent knowledge). The mean level of knowledge for the sample was 2 and the median was 3.

### Recruitment

To take part in the focus groups, participants were required to meet three selection criteria. They had to be aged 18 years or over, fluent in English, and identifying as lesbian or gay. The sample was recruited purposively through a mix of advertising and snowballing. Advertisements were placed on the Web (eg, Facebook, gay and lesbian-related websites, and the La Trobe University website), in print media (eg, magazines that targeted lesbians and gay men), and on a free-to-air radio station that targets lesbians and gay men in Melbourne, Australia. Advertisements were also included in the newsletters of gay and lesbian support organizations. All advertisements directed prospective participants to a registration website where they provided their contact details and confirmation that they met the selection criteria. In total, 111 eligible prospective participants registered their interest. The first author divided registrants into the four demographic categories (see Procedure section), from which he randomly invited registrants to focus groups, with the aim of having approximately four participants per group. Reimbursement of A$30 was offered for participation.

### Procedure

This study received ethics approval from La Trobe University Human Research Ethics. The focus groups were held at the Australian Research Centre in Sex, Health and Society at La Trobe University in Melbourne. Eight focus groups were conducted, comprising two groups for each of the following four demographic categories: (1) younger lesbians aged 18 to 34 years (YF), (2) younger gay men aged 18 to 34 years (YM), (3) older lesbians aged 35+ years (OF), and (4) older gay men aged 35+ years (OM).

These four categories were chosen in light of research that shows key differences in the mental health and experiences of sexuality-related stigma among same-sex attracted people according to age [32,33] and sexual identity [34]. An age limit of 35 years was set for the younger group to account for many early-life milestones that may have recently been experienced by this group and can be potential sources of stigma-related challenges, such as coming out for the first time, gaining a higher education, entering the workforce, and establishing long-term relationships. Limiting the number of focus groups to eight was deemed appropriate, as similar ideas were expressed between all groups, thus suggesting that additional focus groups were unlikely to result in further substantive data. Focus groups were conducted between October and November 2013. Each group took up to 90 minutes and was digitally audio-recorded.

The aim of the focus groups was to evaluate the needs and experiences of lesbians and gay men in the provision of depression and anxiety e-therapies. To facilitate discussion, participants were asked to examine a series of case studies drawn from the Australian e-therapy “MoodGYM”, which follows the lives of six characters as it takes users through a structured therapeutic program comprising reading, exercises, and evaluative tools [35,36]. Excerpts from MoodGYM were chosen as stimulus material because it is one of the most prominent e-therapies [37], has been repeatedly proven to be clinically effective, and is included in the Substance Abuse and Mental Health Services Administration (SAMHSA) Registry of Evidence-Based Programs and Practices [38], and its material typifies features found in many prominent e-therapies, such as the use of characters as examples, a CBT modality, and interaction with users’ everyday experiences as part of therapy.

All focus groups were facilitated by the first author, who is male, and has prior training and experience in conducting focus groups with men and women. At the beginning of each focus group, participants were briefed by the facilitator about his research background, key information about e-therapies, and mood disorders. The facilitator did not disclose his sexual orientation to the focus groups. Each focus group was then presented with the following printed MoodGYM case studies, in order: a set of character profiles and stories/examples that featured the characters; a questionnaire designed to identify dysfunctional beliefs and attitudes related to relationships and other life experiences, known as the Warpy Thoughts Scale [39]; and excerpts from a module that addresses mood problems related to romantic relationships, known as the Relationships Module. For each case study, participants were asked to discuss the material with regard to its applicability to lesbians and gay men. Additional topics were also discussed that did not require excerpts from MoodGYM, such as whether referrals were needed to mental health services that explicitly targeted same-sex attracted people. After discussing MoodGYM, participants were then given opportunities to discuss content they thought should be included in e-therapies more generally.

### Analysis

The focus group discussions were first transcribed using an independent transcription service. All transcripts were subsequently checked for accuracy by the first author. We then conducted a thematic analysis of the data [40]. The first two authors of this article were the researchers who were primarily responsible for the analysis. Both researchers first read the transcripts independently and then met to discuss the main themes they had identified. Both researchers had arrived at similar broad themes. Following discussion, the first-named researcher developed a more detailed coding system, which informed the final analysis. The first-named researcher then coded all transcripts in detail, and codes were recorded in a spreadsheet. The first-two named researchers met regularly throughout the coding process to review the coding system and to discuss emerging themes. Through this process, four main themes were identified. These were: making e-therapies more inclusive; making e-therapies more relevant to stigma-related challenges faced by lesbians and gay men, including vulnerable subpopulations; and preferences for delivering tailored content. In this article, we report on the main themes and have included quotes to further illustrate key aspects of each theme. The first two named researchers met regularly to discuss the choice of quotes and to ensure that those selected were consistent with the data they represented, were typical of comments made by participants in the focus groups, or provided further relevant detail. Because data was collected in focus groups, it was not always possible to identify the voices of individual participants in audio recordings. The quotes presented are therefore identified according to the group, such as “YF” to indicate a quote from a younger lesbian focus group, rather than according to individual participants. The remaining authors of this article scrutinized the final analysis, including the choice of quotes, and suggested further refinements to guide the interpretation of the findings. For this analysis, participants did not provide feedback on the findings.

## Results

### Overview

Although we divided focus groups into four demographic groups (as outlined earlier), the groups generally gave similar feedback and suggestions. Unless otherwise specified, all of the findings reported below should be read as applying across the focus groups and not limited to particular age or sexual identity categories.

### Making E-Therapies More Inclusive

All focus groups indicated that it was important for e-therapies to acknowledge and represent lesbians and gay men. One of the ways in which participants felt excluded from the MoodGYM examples was through the language used. Focus groups suggested that it would be better if neutral language was used, for example avoiding words like “spouse” and using words like “they” instead of “he” or “she”. Focus groups also suggested that examples, such as images, stories, or scenarios, needed to include same sex-attracted people to be relevant:

It’s about being included and it’s about identifying with the character, and the specific experience of being lesbian is very different from that of a straight woman or a gay man or a heterosexual man. It’s important to be represented.

OM

It’s heterosexual and I don’t relate to it.

OF

Groups largely did not support the cast of MoodGYM characters, which they felt did not represent non-heterosexual issues. They had varied views as to how best to achieve a representative cast of characters. Suggestions ranged from using a cast of mixed-sexuality characters to using androgynous characters:

I would probably either go with… a mix of gay and straight [characters], or just random men and women that go on dates with people, with androgynous partners.

YF

[Androgynous] would work for me in certain situations. But I think a lot of people would also like to identify with the person… some people would rather have that kind of personalized connection.

YM

I think I would want at least fifty percent to be not heterosexual, like I know that’s not in line to statistics in society or whatever but to feel like it wasn’t just the stereotypical one or token one or whatever… I’d feel like you’d need quite a breadth of representation.

YM

Focus groups also suggested that inclusiveness could be improved by making lesbians and gay men feel welcome at the beginning of the e-therapy program by explicitly mentioning them upon sign-up:

I’m thinking that in terms of this service, the e-therapy, from the initial, if they use more inclusive terminology and things… that might help to improve the uptake.

OF

Another participant in that group added:

It’s an enormous first step up. It’s like you respect me and you’re going to treat me and help me... So if you’ve included me on the form [referring to early in the e-therapy], therefore you care.

OF

Including a rainbow flag, which is a universal indicator of a gay and lesbian inclusive service, was one further suggestion. However, focus groups were reticent about specifically singling out sexuality at the beginning of an e-therapy that is aimed toward general users, instead preferring a broader statement of inclusiveness that included sexuality alongside gender, ethnicity, and other diverse populations.

Though suggestions about how to achieve inclusiveness varied, a consistent underpinning theme for all focus groups was that inclusion is needed. Avoiding language and content that suggests or assumes users are heterosexual was a major theme in all focus groups, and having some direct references to lesbians and gay men, such as including a gay character in examples and stories, was a common preference.

### Making E-Therapies More Relevant to Lesbians and Gay Men

Addressing stigma and stigma-related issues, such as coming out, were the most prominent type of content requested by focus groups for making e-therapies more relevant to lesbians and gay men. This included content to assist users to manage and address public stigma, such as discrimination and prejudice, as well as self-stigma, such as experiences of shame or having negative beliefs and attitudes toward their own sexuality [41,42]. Groups recurrently flagged both kinds of stigma as a cause of mood disorder issues, tended to react favorably to MoodGYM examples that they perceived as relevant to addressing stigma, such as some items in the Warpy Thoughts Scale, and made numerous suggestions for content to be added to e-therapies that targeted stigma-related issues. With regard to public stigma, focus groups flagged several forms of stigma that they suggested should be addressed in e-therapies:

Homophobia manifests itself in so many ways, and there’s so many kinds of homophobia, and you know there’s overt homophobia, there’s covert homophobia, there’s implied homophobia, there’s homophobia that happens when no one intends to be homophobic at all… I think a lot of the things that cause depression and anxiety in the gay community are linked to one of those many kinds of homophobia that’s out there.

OM

Just being part of a society that is overall really heteronormative and you know like constantly not being part of things and not being necessarily discriminated against as such but like finding lack of representation or just like kind of coming up against things that don’t fit you or your family or your friends or the way that you live, that sort of thing.

YF

A number of focus groups suggested that e-therapies needed to provide a safe space from stigma in order to be effective:

I think if you want something like this to work, you have to provide a forum where people feel like they’re in a comfortable environment, and you know I mean a lot of people want some respite from the straight world, and they’re particularly going to want that when they’re dealing with issues to do with mental health.

OM

Coming out was a key issue. Groups indicated that coming out should be addressed as an ongoing process of deciding whether to disclose or conceal, or “coming out” and “coming in”, as the Working Therapeutically with LGBTI Clients: A Practice Wisdom Guide has termed it [43]:

You’re coming out every day; always deciding if it’s safe to come out.

YM

You often need to hide your relationship, which would be good to cover [in e-therapy].

YM

With regard to internalized stigma, groups also expressed a need to address issues of shame and negative self-perceptions:

I’ve definitely felt that [internalized homophobia] and I think that, you know, if there was more focus on you know accepting who you are, and you know ways to develop yourself that are outside of, you know, just getting a partner or getting a relationship or whatever then that would be a good way to go I think.

OF

Internalized homophobia is a big issue… you can catch yourself every now and then thinking ‘oh well if I wasn’t gay I would be doing this, I would be better and I would be’; it’s… how we think how other people see us... And it comes down to just having enough self-worth. Say well… this is me and I am getting on with my life. That’s all I would ever want.

OM

Participants also highlighted a range of issues that arose from within gay and lesbian communities, and portrayed these communities as sources of stigma and exclusion as well as sources of strength and resilience. This reflects previous research that identifies subcultural groups within gay communities that have potential for marginalization within these communities [44]. Older age, especially among men, can also be stigmatized within the gay community [32,33,45]. Thus, e-therapies may need to avoid addressing stigma as an issue that only comes from outside gay and lesbian communities:

I think [ageing] that’s one of the biggest issues of all… You know, it’s sort of like people are just, ‘oh you are only an old poof’, this sort of business. You know it’s a throwaway thing but it’s bullying and it hurts.

OM

In response to MoodGYM’s relationships module, focus groups commented on the lack of relevance to lesbian and gay relationships:

It has to be specific, I mean if I was to go online for relationship breakup assistance, and I was to see an icon of a man and an icon of a woman, I would just think this is aimed at straight people. It’s going to have no concept of same-sex relationships, or issues, or dialogue at all, so what’s the point?

OM

They specifically commented on the large attention given to breakups and tensions within relationships that characterized examples presented within the relationships module. While relevant, focus groups pointed out the lack of attention given to the pressures faced in a healthy but stigmatized relationship, and suggested content and support to address these additional pressures:

It’s tough to be seen holding your girlfriend’s hand in public... you kind of feel constantly on edge in public… It’s not the relationship itself; it’s the context that it occurs in. And the context is hostile a lot of the time.

OF

Surprised there’s just breakup; there’s as much anxiety about starting a relationship. There should be [more than just breakup].

YM

Groups further stressed that while the issues they face in the breakup of a relationship are fundamentally the same as for everyone else, they are faced with additional challenges related to the acceptance of their relationships, such as from family:

Family should be covered. Aren’t I getting married? When can we expect grandchildren?

OF

My family, they don’t view it as a real relationship, to them it’s nothing.

OF

To further improve the relevance of e-therapies, focus groups also suggested a need for e-therapies to include helplines that cater to the specific needs of lesbians and gay men, and provide a safe space for talking about mental health issues:

Yes, [helplines] must be tailored. There’s extra issues. You need someone who understands what you’re going through. It’s duty of care to provide such resources.

OF

[Helplines] should be targeted, and more available. Perhaps online chat would be good.

YM

While including tailored telephone helplines was the most common suggestion, focus groups pointed to other resources such as websites or referrals to face-to-face services that specialize in the mental health of same-sex attracted people. In particular, younger groups highlighted the positive role that online communities have had in breaking down a sense of isolation and in creating support networks, thus further highlighting a need for e-therapies to provide tailored referrals.

### Including Vulnerable Subpopulations

When discussing the merits of tailoring e-therapy to lesbians and gay men, focus groups suggested a need for therapies to be inclusive and relevant to several subpopulations. Age was a commonly discussed factor. In particular, the younger focus groups suggested a need for e-therapies to give special attention to young people while the older groups mentioned the importance of addressing aging-related issues. Pressures around bullying at school, coming out, and developing an identity were mentioned as important issues for young people, while the death of a partner and finding gay-friendly aged care facilities were mentioned as issues to address for older people. Rural lesbians and gay men are at greater risk of mental health problems [46] and these populations were also flagged by the focus groups as needing specific attention; the pressures of “being the only queer in the village”, as a young female put it. Some participants saw particular value in Web-based modes of communication in reaching these populations:

Coming from the country where I was the only open person in a 60 kilometer radius. And I know that, and not because of Grindr [a mobile phone app that facilitates dating among same-sex attracted men], but because I knew everyone in a 60 kilometer radius. And so it was kind of like having the online resources and social media, you know, connecting with people.

YM

Focus groups also suggested that e-therapies take into account lesbians and gay men from ethnic minority backgrounds. A common point was that some of those with ethnic minority backgrounds face additional challenges in gaining acceptance from others, particularly if they are from traditional or religious cultures. Gaining acceptance within gay and lesbian communities was also flagged as a challenge for some. More generally, those with an ethnic minority background wanted to feel included in e-therapies:

My ethnic background is very tightly aligned with my difficulties with my sexuality and so if I were to see a white female who was same-sex attracted [as e-therapy content], I’d feel like there were a lot of exceptions to me with my case and it might not be a very good tool.

YF

### Preferences for Delivering Tailored Content

When asked about how best to achieve inclusiveness and relevance, all focus groups suggested that some degree of tailoring was required. Many wanted separate e-therapies that were specifically dedicated to lesbians and gay men:

The generalist approaches that works for people, with the gay and lesbian add-on bits, based on what comes out of your research, could be just fine, but I think something specifically tailored would be better.

OM

I’d be more inclined to use the specific one, [to which another participant added]: It would be more relevant and accurate. I’d be more trusting of one.

OF

I personally would prefer to use like a specific one if it, like specific to gays and lesbians, if it is linked to me being a lesbian. Like it’s an issue I face because I am a lesbian. But if I am having a separate issue, more general to you know, I don’t know, my everyday life, which is nothing to do with me being a lesbian, then I would opt for the general one.

YF

While tailoring was broadly supported, there was some concern that creating a specific e-therapy for lesbians and gay men could reinforce marginalization by suggesting that lesbians and gay men could not be included within general e-therapies:

If you’re making a totally separate program and separate website then that just kind of enhances the feeling of ‘okay you guys are separate, you guys are different, so we need something special for you’.

OF

I have worked so hard all my life to have equal friends and relationships. I wouldn’t want to go back to ‘John and Betty get married and Chris and Philesco over here and they live there completely separate’… Everybody together is what we’re trying to aim for.

OF

Some focus groups suggested that general e-therapies might instead use adaptive logic in which content on sexuality and sexuality-related issues is directed to a user’s sexual identity:

Why don’t you customize? When you log on you customize your own.

OF

[Tailoring using adaptive logic] That sounds awesome.

YF

Further extending adaptive logic, some focus groups also suggested incorporating the use of avatars: personalized representations of a user. Groups suggested that inclusion and relevance could be enhanced by allowing users to customize their MoodGYM characters to experience e-therapy as a personalized story that responds to their particular identity:

It should be like The Sims and you should be able to create your own character.

YF

That will work for everyone if they have enough kind of menu items to choose from.

OF

In all, whether e-therapies use adaptive logic or separate dedicated e-therapies are developed for lesbians and gay men, some form of tailoring that delivers targeted content for addressing issues specifically faced by lesbians and gay men was strongly supported in all focus groups.

## Discussion

### Principal Findings

Focus group participants offered a range of insights into how e-therapies could be made more applicable to lesbians and gay men. They articulated a need to address stigma-related challenges, including those from within gay and lesbian communities. This encompassed a need for e-therapies to address same-sex relationship issues in ways that take account of challenges such as stigma and rejection of the relationship, lack of support during breakups, and family rejection or misunderstanding. Attention also needs to be given to language, imagery, and examples when making e-therapies more inclusive and relevant to same-sex attracted people. Incorporating content that is inclusive and relevant to particular subpopulations, such as those living in rural areas and from minority ethnic backgrounds, may also deserve consideration. In addition, focus groups pointed to a need for making e-therapies more relevant by including helplines and other resources that provide appropriate follow-up options for lesbians and gay men.

All focus groups expressed a need for e-therapies to involve some form of tailoring to lesbians and gay men. On balance, having e-therapies available that are specifically targeted to non-heterosexual users received the greatest support. However, there was some tension around this. Some participants were concerned that providing separate content for lesbians and gay men draws a problematic segregation between heterosexual and non-heterosexual and may therefore run counter to the principle of inclusiveness. Other participants argued that the needs of lesbians and gay men are significantly different, and that content should therefore be tailored explicitly and specifically in order that e-therapies adequately address the needs of these populations. In fact, having an explicit acknowledgement of lesbians and gay men appears to be particularly important because “lack of acknowledgement” and “invisibility” were mentioned in every focus group and were highlighted as major stressors by participants. A large number of comments in focus groups also referred to a need to be specifically spoken to by e-therapies.

While having separate e-therapies for lesbians and gay men is one option, developers could consider fine-tuning e-therapies aimed at the general population, such as MoodGYM, so that they are also inclusive and relevant to lesbians and gay men. Adaptive logic is one mechanism that can be used to achieve this. Adaptive logic presents different content to users based on their responses to questions asked in the e-therapy. Adaptive logic is already a common feature of e-therapies, but is presently not being used to tailor e-therapies for different sexual orientations. That said, the issue of segregating users also applies here. Thinking about subtle ways of delivering targeted content according to a user’s sexual orientation may be advisable rather than having clearly demarcated areas of an e-therapy for heterosexual and non-heterosexual users.

The various suggestions that emerged from the focus groups should be evaluated in the context of participants responding favorably to many aspects of the MoodGYM case studies. The request to provide extra content to address additional stressors on top of largely effective general content is congruent with experiences described by Minority Stress Theory [21,47], which is currently the dominant model to account for the mental health implications of living with a stigmatized identity. Minority Stress Theory proposes that stigmatized populations often withstand additional stress derived from their minority status on top of general life stress that people of all backgrounds may face. Some suggestions, like including references to helplines specific to same-sex attracted people, are low-cost and easy to implement improvements. Others, like the delivery of targeted content on stigma-related stress, are more complex. However, on the whole, we believe that the feedback offered in our focus groups, read in the context of broader recommendations for tailoring mental health strategies to non-heterosexual people, may prove useful for e-therapy developers.

A paper by Lucassen et al [11] is, to our knowledge, the only other published work that qualitatively assesses the views of same-sex attracted people on tailoring computerized CBT programs (cCBT), although it relates to a CD-ROM program, “SPARX”, rather than an e-therapy. Lucassen et al identified coming out and other stigma-related challenges such as obtaining family support as major problems faced by their sample, and also identified coping mechanisms, including social support. Their sample also responded positively to a “rainbow” version of the SPARX program, tailored to same-sex attracted people. Major themes and suggestions offered by participants in our study correlate closely with those identified by Lucassen et al. Furthermore, suggestions made by our focus groups fit closely with findings from other research that examined the experiences of same-sex attracted people within broader health care services. For example, issues around non-inclusive language and assumptions about relationship structures that do not account for same-sex attracted people are some of the key areas highlighted in previous research on health care services as problems needing to be addressed [43,48-50], and that also emerged in our focus groups.

Suggestions from our focus groups on how to improve e-therapies are also consistent with broader work aimed at adjusting mental health care for non-heterosexual clients. For example, major policy documents about mental health care, such as those from Stonewall and the UK Department of Health [14,15], and “Going Upstream: A framework for promoting the mental health of lesbian, gay, bisexual, transgender and intersex (LGBTI) people” [28] from Australia, all fundamentally advocate for tailored responses for same-sex attracted people. Recommendations in these documents are closely related to themes and suggestions that emerged in our focus groups, such as the need to address stigma-related challenges, to specifically address same-sex relationships, to account for the needs of vulnerable subpopulations, and to provide relevant referral services.

### Limitations and Future Directions

Our sample was highly educated compared to the general Australian population. Education is strongly linked to socioeconomic status, and there is potential for the feedback we received from the focus groups to reflect the ideas and concerns of a higher socioeconomic group. Notably, many participants seemed to already be engaged in broader debates related to lesbian and gay mental well-being and entered our discussion with a depth of existing perspectives to draw on. Future work may need to be conducted with lower socioeconomic groups, as access to mental health services and experiences of stigma-related stress may be different.

Another possible limitation is that data about the participants’ history of mood disorders was not sought. The topics discussed were about e-therapies’ relevance to the needs and experiences of lesbians and gay men, and it was important to understand how this can best be achieved for the lesbian and gay population in general. Therefore, it would not have been optimal to target only those with previous experience of depression or anxiety. However, it is possible that participants with a history of mood disorders may have had different perspectives on the topics discussed, and future studies may wish to take this into account. Researchers who conduct future studies that are similar to our study may also wish to consider the potential role of ethnicity. We did not collect data on the ethnic backgrounds of participants, but the intersection of ethnic and sexual identities was raised as an issue by participants in our study. Having data on the ethnic backgrounds of participants may therefore be useful for further contextualizing the findings that emerge from future research.

Focus groups were also limited to a discussion of MoodGYM rather than other specific e-therapies. Decisions about which parts of MoodGYM were used as case studies were undertaken with the aim of generating findings that could be generalized to other e-therapies. Furthermore, we believe MoodGYM to be a good prototype for our study because it is prominent, well-evidenced, and is often regarded as a benchmark for other e-therapies. It also uses “scenarios”, which are commonly found in well-evidenced e-therapies, but can make it challenging to incorporate content that is inclusive of diverse populations [29]. Nevertheless, our findings may have been a little different had another e-therapy been used as a case study, and any generalization of our findings to other e-therapies entails a degree of speculation.

Finally, we acknowledge that the scope of this study was limited by encompassing only lesbians and gay men. The exclusion of other non-heterosexual groups, as well as sex and gender-diverse groups (such as intersex, gender questioning, and transgender people), was decided because each group has different health outcomes and often has different life experiences to those of lesbians and gay men [30,31]. For example, bisexual men and women sometimes report feeling excluded from both heterosexual and gay communities. Thus, it would be inappropriate to lump all of these groups together, and conducting separate focus groups for each group was beyond the scope of this study. It is therefore recommended that future studies build on our work to encompass other sexual and gender identity groups. Some of the specific suggestions from this study may not necessarily be applicable to these other groups, but the general themes around improving inclusiveness and relevance are still likely to apply.

### Conclusions

At present, e-therapies for depression and anxiety fail to cater to the mental health needs of lesbians and gay men, and many actively exclude lesbians and gay men by delivering content that assumes or suggests users are heterosexual. Through a series of focus groups, this study aimed to identify key areas in which e-therapies might be improved. Focus groups raised a number of issues that broadly related to improving the inclusiveness and relevance of e-therapy, such as avoiding language and content that excludes same-sex attracted users, addressing stigma-related challenges by expanding content on relationships, coming out, managing discrimination, and coping with lack of support from family and friends, as well as providing additional resources that specifically cater to same-sex attracted people. Our findings give further weight to broader advocacy and policy work aimed at improving mental health resources and health care for lesbians and gay men. Our findings also build on previous research, such as a recent protocol offered for improving e-therapies for lesbians and gay men [29] and the qualitative work by Lucassen et al [11]. Taken together, findings from both this study and previous work provide e-therapy developers with guidance to improve the inclusiveness and relevance of e-therapy for lesbians and gay men, and to help strengthen the role of e-therapy in reducing high rates of depression and anxiety among these populations.

## Abbreviations

CBT: cognitive behavioral therapy
CCBT: computerized cognitive behavioral therapy
OF: older female focus group
OM: older male focus group
YF: younger female focus group
YM: younger male focus group
